# Clinical significance of dysregulation of miR-381 in pediatric acute myeloid leukemia

**DOI:** 10.1186/s40001-020-00442-1

**Published:** 2020-09-16

**Authors:** Piqiang Zhang, Deyun Sun, Xuemei Sun, Hongjuan Li

**Affiliations:** Department of Pediatrics, Linyi People Hospital, No. 27, East Jiefang Road, Linyi, 276003 Shandong China

**Keywords:** miR-381, Prognosis, Diagnosis, Acute myeloid leukemia

## Abstract

**Background:**

microRNA-381 is dysregulated in a variety of cancers. However, its clinical significance in pediatric acute myeloid leukemia (AML) is still unclear. The purpose of this study was to detect the expression level of miR-381 in pediatric AML patients and to explore its potential clinical significance.

**Methods:**

The levels of miR-381 in bone marrow and serum of 102 pediatric AML patients were measured by quantitative real-time polymorperase chain reaction (qRT-PCR). The diagnostic value of serum miR-381 in pediatric AML patients was evaluated by the receiver operating characteristic (ROC) curve. A Chi square test was used to analyze the relationship between serum miR-381 and the clinical characteristics of patients. Cox regression analysis and Kaplan–Meier evaluated the prognostic value of serum miR-381 in patients. Finally, the proliferation of the cells was analyzed by the CCK-8 assay.

**Results:**

Compared with healthy controls, the levels of miR-381 in serum and bone marrow of pediatric AML patients were significantly decreased (*P *< 0.001). ROC curve showed that miR-381 could distinguish pediatric AML cases from normal controls. At the same time, the downregulation of miR-381 was associated with M7 in the French–American–British (FAB) classifications and unfavorable cytogenetic risks (*P *< 0.05). Low serum miR-381 levels were associated with poor overall survival of pediatric AML (log-rank test, *P *= 0.011) and poor relapse-free survival (log-rank test, *P *= 0.004). Cox regression analysis confirmed that reduced serum miR-381 was an independent predictor of poor prognosis in AML (HR = 3.794, 95% CI 1.3633–10.559, *P* = 0.011). In addition, low expression of miR-381 significantly reduced the proliferation of cells (*P *< 0.05).

**Conclusion:**

All experimental results confirm that miR-381 has reduced bone marrow and serum expression in pediatric AML, and low levels of serum miR-381 have certain diagnostic and prognostic value in pediatric AML and may be a potential therapeutic target for AML.

## Background

Leukemia has the highest incidence of all types of childhood cancer, and its incidence continues to rise [1]. Acute myeloid leukemia (AML) is a clonal disease of hematopoietic tissue produced by malignant transformation of bone marrow-derived, self-renewing stem cells, or myeloid progenitor cells [2]. AML is characterized by blocked differentiation and abnormal proliferation, which can lead to bleeding, fatal infection, or organ infiltration [3]. Pediatric AML accounts for 20% of pediatric leukemia [4]. Despite significant advances in improving the treatment of AML over the past few decades, adult and pediatric AML respond differently to treatment and prognosis, and AML continues to threaten the lives of children. The overall survival rate for childhood AML is only 70% [5, 6]. Therefore, it is necessary to explore new biomarkers for the diagnosis, prognosis, and therapeutic targets of pediatric AML in order to develop more effective surveillance and treatment regimens.

MicroRNAs (miRNAs) are non-coding RNA molecules composed of 19–22 nucleotides that can play a critical role as oncogenes or tumor suppressor genes in the development and progression of multiple solid or blood tumors, including AML. For example, miR-10a/b is abnormally highly expressed in AML patients and promotes the proliferation of promyelocytic leukemia [7]. In addition, due to its high conservatism, stability, sensitivity, and extensive presence in tissues and body fluids (blood, saliva, plasma, and serum), miRNAs have been widely studied as biomarkers for clinical diagnosis and prognosis of a variety of diseases.

What is interesting is that the nucleic acid analog cytarabine is a major component of chemotherapy for AML. Bhise et al. [8] identified miRNAs involved in the regulation of mRNA expression levels of cytosine arabinoside pathway genes in multiple AML cell lines, including miR-381. miR-381 is located on the human chromosome 14q32.31 and has been reported to be involved in the progression of tumors, including gastric cancer, breast cancer, and colorectal cancer [9–11]. At the same time, the change of miR-381 can regulate the multidrug resistance in leukemia cells [12]. In addition, serum miR-381 has been reported as a non-invasive biomarker for the diagnosis and prognosis of thyroid papillary carcinoma [13]. However, the clinical value and biological role of miR-381 in pediatric AML have not been investigated.

The purpose of this study was to evaluate the expression and clinical value of serum miR-381 in pediatric AML, as well as its regulatory effect on cellular biological behavior. Our data suggest that miR-381 may be a novel prognostic and diagnostic marker and a potential molecular therapeutic target for pediatric AML.

## Materials and methods

### Clinical samples

This study was approved by the medical ethics committee of Linyi People Hospital, and parents or the patients provided informed consent. All specimens were anonymized in accordance with ethical and legal standards.

102 newly diagnosed pediatric patients with AML were retrospectively selected from Linyi People Hospital. There were 52 male and 50 female patients, all of whom were under 18 years old. All AML patients were diagnosed using Wright Giemsa staining smears and morphological evaluation of immunophenotypes by flow cytometry. According to the French–American–British (FAB) classification system, of the 102 cases 1 was AML M1, 38 AML M2, 3 AML M3, 25 AML M4, 19 AML M5, 4 AML M6, and 12 AML M7, respectively. All patients were classified for the cytogenetic risk status according to the current National Comprehensive Cancer Network AML guidelines: the favorable-risk patients were referred to those harboring karyotypic abnormalities with inv(16)/t(16;16), t(15;17) or t(8;21); intermediate-risk patients comprised those patients with normal cytogenetics, +8, t(9;11) or other nondefined karyotypes; and the unfavorable-risk patients were those with karyotypes including complex (three clonal chromosomal abnormalities), -5, 5q-, -7, 7q-, 11q23 - non t(9;11), inv(3), t(3;3), t(6;9), and t(9;22) [14]. Patients received 10 days of induction chemotherapy, and the dose of chemotherapy was adjusted according to the bone marrow response on day 7. The characteristics of the 102 AML cases were recorded in Additional file 1: Table S1. All patients were followed regularly for 60 months, and their overall survival (OS) was defined as the time from the initial diagnosis to death. Relapse-free survival (RFS) is defined as the time from the complete remission to recurrence. In addition, 50 healthy children of similar age were selected as the control group in this study, who had no clinical symptoms of cancer, liver, joint, metabolic or endocrine diseases, and whose bone marrow morphology was detected by cytology and histology. 5 mL of venous blood from each subject was extracted, centrifuged after static treatment, and serum was collected and stored at − 80 °C.

### Cell culture and transfection

Human bone marrow stromal cells HS-5 and AML cell lines THP-1 and HL-60 were purchased from American Type Culture Collection (ATCC, Manassas, VA, USA). All cells were maintained in RPMI-1640 medium (Invitrogen, Carlsbad, CA, USA) containing 10% fetal bovine serum (FBS) and 1% penicillin–streptomycin reagent, and cultured in a humidity incubator at 37 °C and 5% CO_2_. miR-381 inhibitor, miR-381 mimic, and their negative control of non-coding miRNAs (inhibitor NC and mimic NC) were purchased with RiboBio (Guangzhou, China). Their sequences were as follows: miR-381 mimic, 5′-UAUACAAGGGCAAGCUCUCUGU-3′; mimic NC, 5′-UUCUCCGAACGUGUCACGUTT-3′; miR-381 inhibitors, 5′-ACAGAGAGCTTGCCCTTGTATA-3′; inhibitor NC, 5′-CAGUACUUUUGUGUAGUACAA-3′. The cells were seeded into a 6-well plate overnight. Transfection reagent Lipofectamine 2000 was used to regulate the expression of miR-381 in vitro when the cells reached the logarithmic growth stage. Change the fresh medium after 6 h according to the instruction. After 48 h, the cells were used for further experiments. The untreated cells were used as control group.

### RNA extraction and qRT-PCR analysis

In order to evaluate the expression level of miR-381 in the serum and AML cell lines of patients, real-time fluorescence quantitative RT-PCR was used to detect miR-381 in this study. Total RNA was extracted from serum using QIAamp RNA blood kit according to manufacturer protocol. miRNA purification kit (Cwbiotech, Beijing, China) was used to extract the total RNA from cell lines. The extracted total RNA was produced by reverse transcription reaction using a miRNA cDNA synthesis Kit (Cwbiotech, Beijing, China). Subsequently, a quantitative RT-PCR reaction was performed on the ABI 7500 real-time PCR system through the miRNA qPCR Assay kit (Cwbiotech, Beijing, China). During the reaction, miR-39 was used as endogenous control, and the relative expression of miR-381 was calculated by 2^−ΔΔCt^.

### Cell proliferation assay

CCK-8 assay was used to detect cell proliferation ability. After transfection with miR-381 mimics and inhibitors, 2.0 × 10^3^ were collected and seeded into a 96-well plate. Cell proliferation was detected at every interval of 24 h. Before detection, 10 μl CCK-8 reagent was added to the cells and then incubated in the incubator for 1 h. The absorbance value at 450 nm was detected on the microplate reader. Each group had three well at each time point, and the experiment was repeated three times.

### Luciferase reporter assay

TargetScan (v7.2) bioinformatics software (http://www.targetscan.org/vert_72/) was used to predict target genes of miR-381, then the luciferase reporter assay was performed to verify the results. The 3′-untranslated region (UTR) of high mobility group box 1 (HMGB1) was cloned into the luciferase reporter vector psiCHECK-2 (Promega Corporation) according to the manufacturer’s instruction. Then 500 ng of each reporter construct (wild-type (WT) or mutant 3′-UTR of HMGB1) and miR-381 mimic or inhibitor were co‑transfected into THP-1 cells by using Lipofectamine 2000. The relative luciferase activity was measured by Dual-Luciferase Reporter System (Promega Corporation, USA) according to the instructions of the manufacturer. Renilla fluorescence activity was identified as the internal reference.

### Statistical analysis

SPSS 21 software and GraphPad Prism 7 software was used for data statistical analysis. The statistical differences between the two groups were analyzed using the student’s *t* test, and one-way analysis of variance (ANOVA) was used to evaluate the differences beyond the two groups. The data were presented as the mean ± SD. Receiver operating characteristic (ROC) curve and area under the curve (AUC) were used to evaluate the diagnostic value of serum miR-381 in pediatric AML. *χ*^2^ test was used to analyze the relationship between serum miR-381 and the clinicopathological features of patients. Kaplan–Meier survival curve was used to determine the relationship between serum miR-381 level and OS or RFS. *P* < 0.05 was considered statistically significant.

## Results

### Decreased expression of miR-381 in patients with pediatric AML

**Fig. 1:**
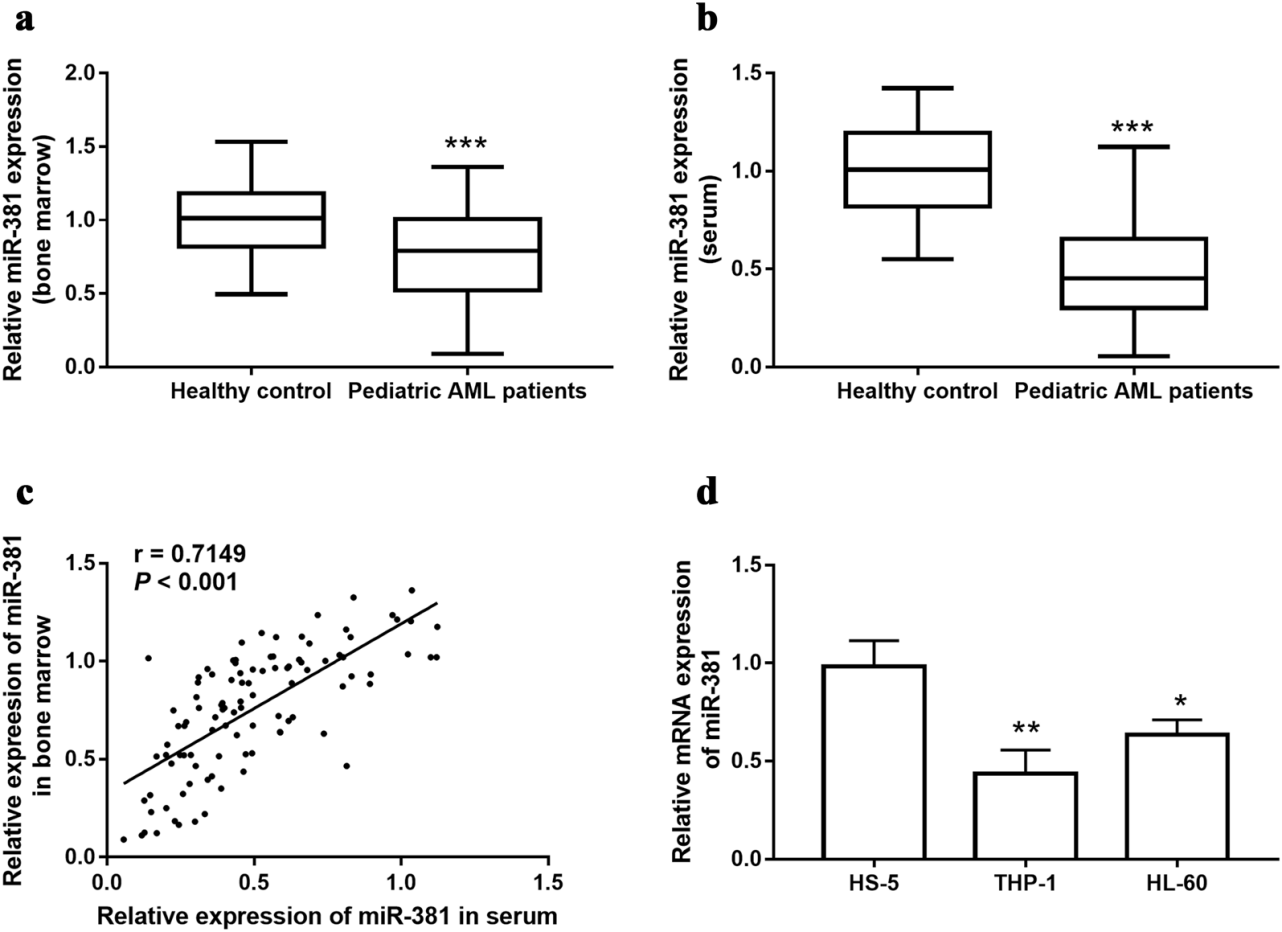
The expression of miR-381 was decreased in pediatric AML patients. **a** Compared with the healthy control group, the expression level of miR-381 in pediatric AML bone marrow was significantly reduced. **b** Compared with the healthy control, the level of miR-381 in the serum of pediatric AML was significantly reduced. **c** The serum level of miR-381 in pediatric patients was positively correlated with the level of miR-381 in bone marrow (*r* = 0.7149). **d** Compared with normal human bone marrow stromal cells HS-5, the level of miR-381 in AML cells was significantly reduced. *** *P* < 0.001, compared with Healthy control

To investigate the level of mIR-381 expression in pediatric AML, qRT-PCR was used to analyze bone marrow and serum from 102 patients and 50 healthy control. As shown in Fig. 1a, b, the expression levels of miR-381 in the bone marrow (0.762 ± 0.312 vs 1.010 ± 0.240) and serum (0.496 ± 0.257 vs 1.000 ± 0.238) of the patients were significantly decreased compared with healthy control (*P* < 0.001). It was also noted that the expression of miR-381 in the bone marrow and serum was significantly positively correlated (*r* = 0.7149, *P* < 0.001, Fig. 1c). Since it is more convenient to collect serum samples, we used serum samples to carry out subsequent experiments. In addition, our study also found that compared with the human bone marrow stromal cells HS-5, the level of miR-381 in AML cells THP-1 and HL-60 was significantly reduced (*P* < 0.001, Fig. 1d). This is consistent with the level of expression in the bone marrow.

### Low-expression of miR-381 was correlated with the clinicopathological features of pediatric AML

In order to study the relationship between serum miR-381 level and the clinical characteristics of pediatric AML, patients were divided into the group with high expression of miR-381 (*n* = 40) and the group with low expression of miR-381 (*n* = 62) according to the mean expression level of miR-381 (0.496 ± 0.257). *χ*^2^ analysis showed that serum expression of miR-381 was significantly correlated with the FAB subtyping and cytogenetics (*P* < 0.05, Table 1). However, the expression of miR-381 showed no significant correlation with patients’ age, gender, leukocyte, WBC counts, extramedullary disease, and Day 7 response to treatment (*P* > 0.05, Table 1).

**Table 1:** Characteristics of the 102 patients with pediatric acute myeloid leukemia

Parameters	Cases No. (*n* = 102)	miR-381 expression		*P*
		Low (*n* = 62)	High (*n* = 40)	
Age (years)				0.840
> 6	45	28	17	
≤ 6	57	34	23	
Gender				0.686
Male	52	33	19	
Female	50	29	21	
WBC counts (× 10^9^/L)				
> 10	52	31	21	0.841
≤ 10	50	31	19	
Leukocyte (/μl)				0.316
> 10,000	47	26	21	
≤ 10,000	55	36	19	
FAB classification				0.026
M1–M6	90	51	39	
M7	12	11	1	
Extramedullary disease				0.420
Absent	53	30	23	
Present	49	32	17	
Cytogenetics				
Favorable	17	9	8	
Intermediate	49	24	25	0.010
Unfavorable	36	29	7	
Day 7 response to treatment				
Favorable	62	34	28	0.149
Unfavorable	40	28	12	

### Diagnostic value of serum miR-381 levels in pediatric AML patients

**Fig. 2:**
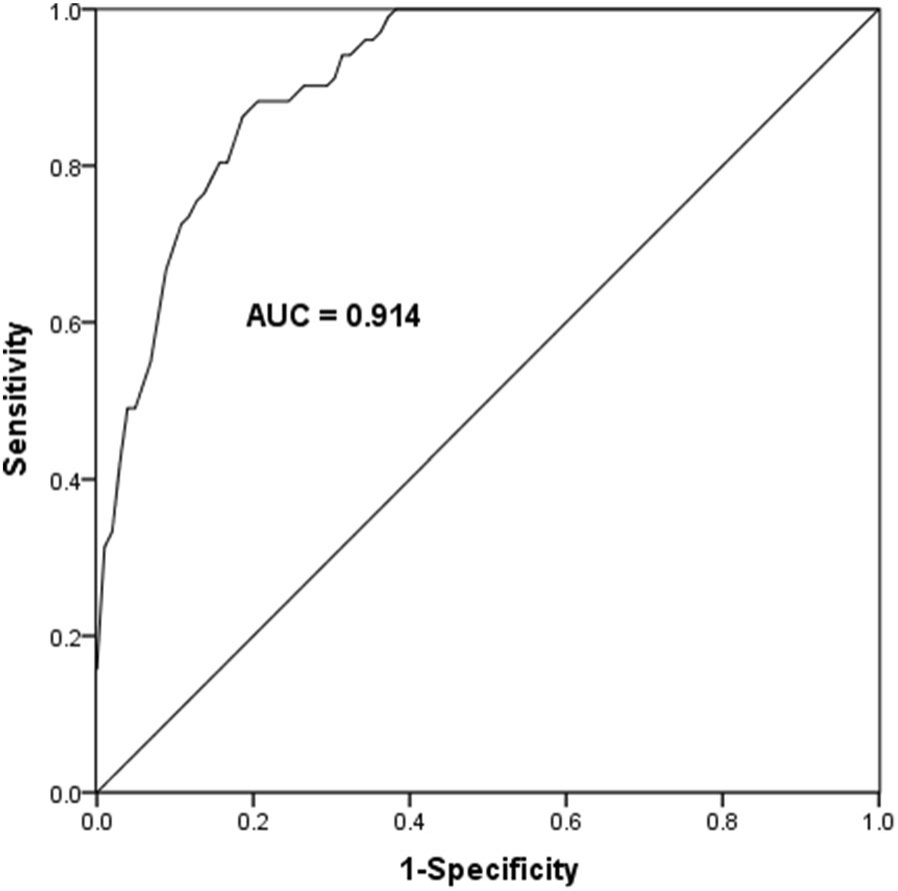
ROC curve evaluated the diagnostic value of serum miR-381 in pediatric AML. The AUC curve was 0.914. When the cut-off value is 0.7515, the specificity and sensitivity are 82.35% and 86.3%, respectively

ROC curve was drawn based on the expression of miR-381 in the serum of patients and healthy control groups to evaluate the diagnostic value of serum miR-381 in pediatric AML. As shown in Fig. 2, the AUC is 0.914, and when the cut-off value is 0.7515, the specificity and sensitivity are 82.35% and 86.3%, respectively.

### Low serum miR-381 was associated with poor prognosis in pediatric AML

**Fig. 3:**
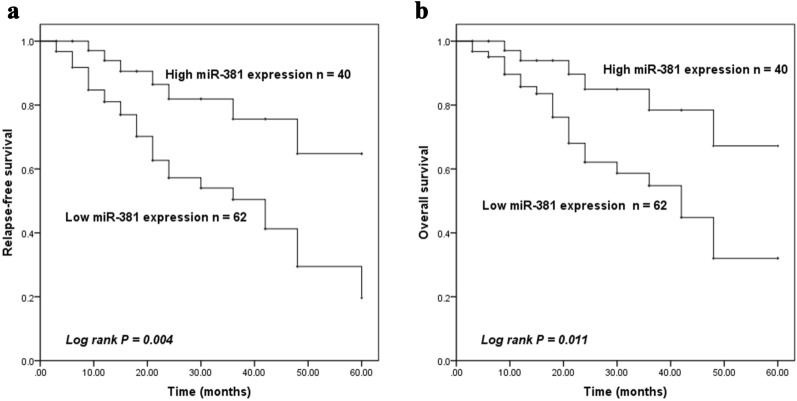
Kaplan–Meier curves of serum miR-381 in pediatric AML patients with relapse-free survival (RFS) and overall survival (OS). According to the mean expression level of miR-381 in pediatric AML patients, all cases were divided into high miR-381 expression group (*n* = 40) and low miR-381 expression group (*n* = 62). Low expression of serum miR-381 in pediatric AML patients had poor RFS (log-rank test *P* = 0.004) and OS (log-rank test *P *= 0.011)

Kaplan–Meier analysis was used to assess the prognostic relationship between miR-381 expression and OS and RFS in pediatric AML patients. According to the mean expression level of miR-381 in pediatric AML patients, all cases were divided into high miR-381 expression group (*n* = 40) and low miR-381 expression group (*n* = 62). As shown in Fig. 3, the overall survival rate of low expression of miR-381 was significantly correlated with the poor prognosis of AML (log-rank test *P* = 0.011). In addition, low expression of miR-381 was significantly associated with RFS in pediatric AML (log-rank test *P* = 0.004). At the same time, Cox regression analysis results showed that miR-381 was an independent prognostic factor for pediatric AML (HR = 3.794, 95% CI 1.3633–10.559, *P* = 0.011, Table 2).

**Table 2:** Multivariate Cox analysis of miR-381 and clinical parameters in relation to overall survival

Parameters	Multivariate analysis		
	HR	95% CI	*P*
miR-381	3.794	1.363–10.559	0.011
Age	0.798	0.348–1.827	0.593
Gender	0.657	0.256–1.686	0.382
WBC counts	0.987	0.446–2.186	0.975
Leukocyte	0.539	0.236–1.234	0.144
FAB classification	3.133	1.230–7.982	0.017
Extramedullary disease	0.682	0.314–1.482	0.333
Cytogenetics	0.513	0.278–0.947	0.033
Day 7 response to treatment	0.783	0.316–1.942	0.598

### Low expression of miR-381 promoted cell proliferation

**Fig. 4:**
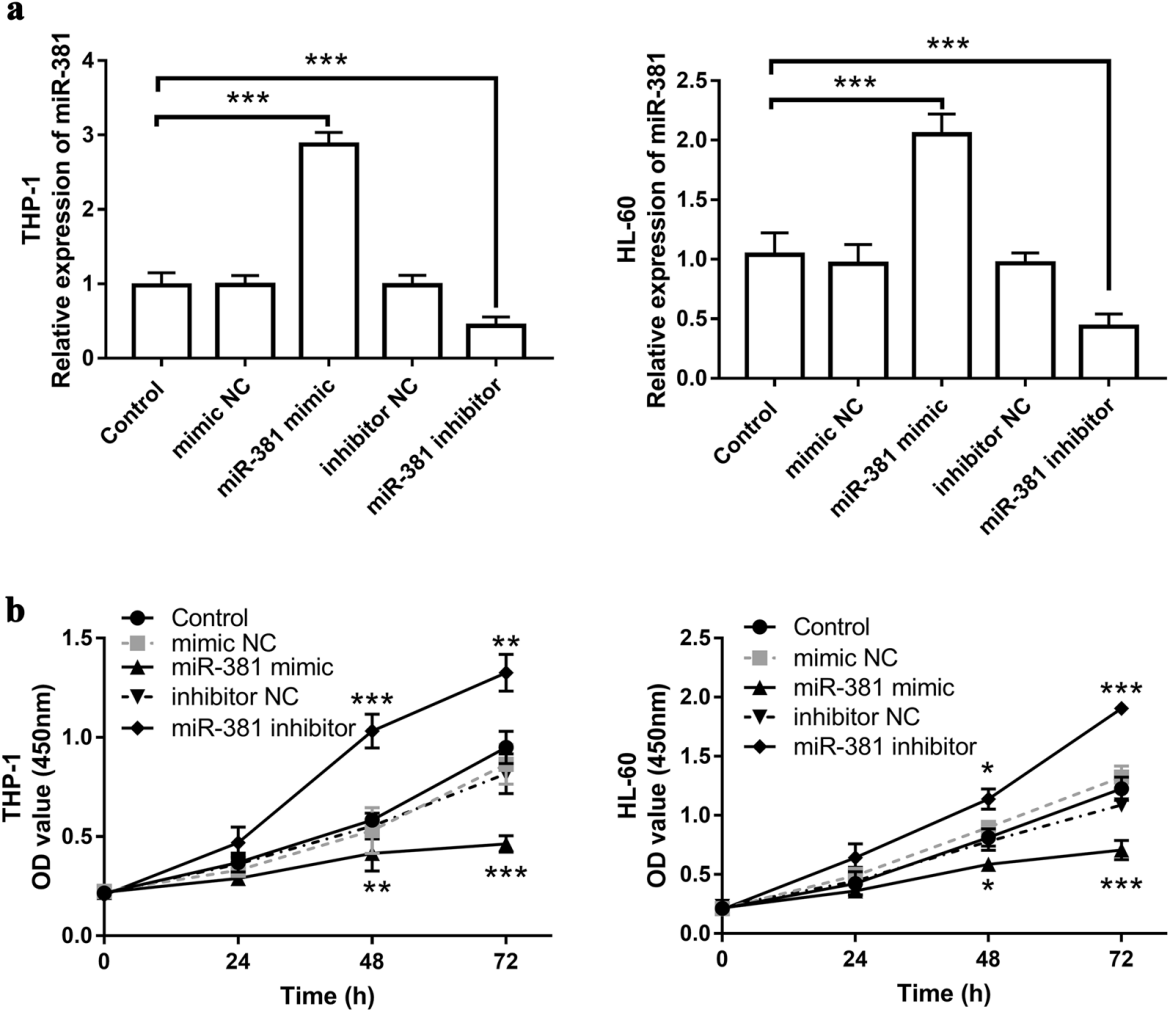
The effect of the miR-381 expression on the proliferation of AML cells. **a** Expression levels of miR-381 after transfection with miR-381 mimic and inhibitor. **b** The effect of miR-381 on cell proliferation was evaluated by CCK-8 assay. Transfection with miR-381 mimic significantly reduced cell proliferation, while transfection with miR-381 inhibitor promoted cell proliferation. ** *P* < 0.01, *** *P* < 0.001, compared with control group. The untreated cells were used as control group

AS abnormal proliferation is one of the characteristics of AML, the effect of miR-381 on the proliferation of AML was finally detected in this study. AML cell lines THP-1 and HL-60 were transfected with miR-381 mimic and miR-381 inhibitor, respectively, and transfection efficiency was demonstrated by qRT-PCR. It was found that compared with the control group, miR-381 was significantly up-regulated by miR-381 mimic and down-regulated by a miR-381 inhibitor (*P* < 0.001, Fig. 4a). After confirming the successful regulation of the expression level of miR-381 in vitro, we examined the effect of miR-381 on cell proliferation. CCK-8 assay showed that miR-381 mimic significantly reduced the proliferation of AML cells compared with the control group, while miR-381 inhibitor promoted the proliferation of cells (*P* < 0.05, Fig. 4b).

### HMGB1 is a direct target of miR-381

**Fig. 5:**
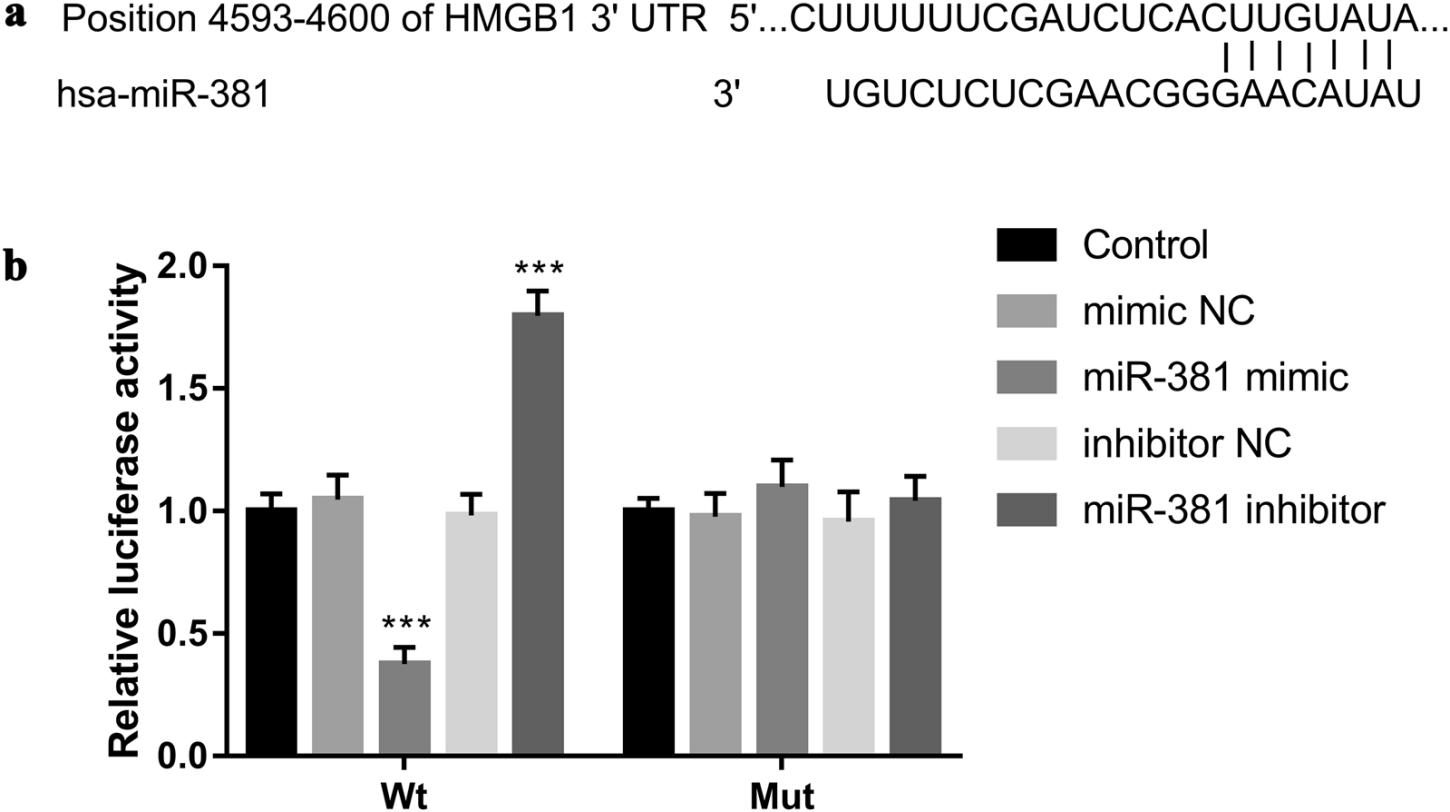
HMGB1 is a direct target of miR-381. **a** Bioinformatics analyze showed that miR-381 contains binding sites for HMGB1. **b** Transfection of miR-381 mimic decreased the luciferase activity in cells transfected with wild type 3′-UTR of HMGB1, whereas the luciferase activity was increased by miR-381 inhibitor transfection significantly. However, mutation in the miR-381 binding sites in the 3′-UTR of HMGB1 abolished the effect on the luciferase activity. *** *P* < 0.001, compared with control group

Bioinformatics analyses showed that miR-381 contains binding sites for HMGB1 (Fig. 5a). Furthermore, the luciferase reporter assay results demonstrated that transfection of miR-381 mimic decreased the luciferase activity in cells transfected with wild-type 3′-UTR of HMGB1, whereas the luciferase activity was increased by miR-381 inhibitor transfection significantly (Fig. 5b). However, mutation in the miR-381 binding sites in the 3′-UTR of HMGB1 abolished the effect on the luciferase activity (Fig. 5b). The results indicated that HMGB1 is a direct target gene of miR-381.

## Discussion

AML is a hematologic malignancy with significant molecular heterogeneity, and nearly 15% of deaths in patients with hematological malignancy are attributable to AML [15, 16]. It is estimated that approximately 150–200 children aged 0–16 years are diagnosed with AML each year [17]. Chemotherapy and stem cell transplantation as the main treatment for AML has improved the prognosis of pediatric AML in recent years. However, pediatric AML patients were less effective in the late stages of metastasis and recurrence. Therefore, how to determine reliable, effective and stable biomarkers for diagnosis and prognosis and effective therapeutic targets remains a major challenge for children with AML. Previous studies have reported that mutations and abnormal expression levels of cancer-related genes may induce AML. Abnormal expression of miRNAs is involved in the development and progression of various human diseases and tumors.

As a potential tumor-related miRNA, miR-381 has been reported to be abnormally expressed in a variety of tumors and involved in cell proliferation, migration, invasion, and metastasis. miR-381 is significantly down-regulated in prostate cancer and regulates cell proliferation and invasion through estrogen receptors [18]. The expression of miR-381 in breast cancer tissues was lower than that in non-cancerous tissues adjacent to cancer and was negatively correlated with cell proliferation, epithelial–mesenchymal transformation, and metastasis [19]. In addition, Chen et al. [20] found that the expression level of miR-381-3p was significantly down-regulated in rats with acute spinal cord injury. It is noticed that Xu et al. [12] found that miR-381 is associated with multidrug resistance in leukemia. In addition, the nucleotide analog cytarabine is a major component of AML chemotherapy, and Bhise et al. [8] identified miRNAs that were significantly associated with nucleotide pathway gene expression in multiple AML cell lines, including miR-381. Therefore, we speculate that miR-381 may play an important role in pediatric AML. Against this background, we first detected the expression level of miR-381, and the results proved for the first time that the expression of miR-381 in bone marrow, serum, and cell lines of pediatric AML was down-regulated. This finding is consistent with studies showing decreased expression of miR-381 in other tumors. In addition, our study also found a significant positive correlation between the expression of bone marrow and serum miR-381.

In recent years, the detection of disease-related miRNAs has attracted people’s attention as a non-invasive disease monitoring tool. The diagnosis and prognosis of miRNAs in pediatric AML patients have been reported [21, 22]. However, miR-381 has been reported as a clinical diagnostic and prognostic marker for a variety of diseases. Low expression of miR-381 is a good prognostic factor that enhances the chemical sensitivity of osteosarcoma [23]. miR-381 is a prognostic factor that inhibits migration and invasion of non-small cell lung cancer by targeting the liver receptor homolog-1 (LRH-1) genen [24]. In order to study the clinical diagnostic and prognostic value of miR-381 in pediatric AML, we used the expression of miR-381 in the serum of patients for subsequent experiments. According to the ROC curve, miR-381 has certain sensitivity and specificity, which can significantly distinguish pediatric AML patients from healthy individuals. The results suggest that miR-381 may be a valuable diagnostic biomarker for pediatric AML. In our study, we also found that the decreased expression of miR-381 was significantly associated with M7 in the FAB classification and with adverse cellular genetic risk. Moreover, pediatric AML patients with low expression of miR-381 had worse RFS and OS. miR-381 was confirmed as an independent prognostic factor for pediatric AML in a multivariate Cox model. The results suggested that miR-381 could also be used as a prognostic biomarker for pediatric AML.

Multiple studies have shown that AML is an uncontrolled clonal proliferation of abnormal myeloid progenitor cells in the bone marrow and blood [25]. Therefore, in our study, we detected the effect of miR-381 on the proliferation of AML cells. After verifying that the expression level of miR-381 can be successfully regulated in vitro, CCK-8 assay confirmed that high expression of miR-381 can significantly inhibit cell proliferation, while the low expression of miR-381 can significantly promote cell proliferation. In addition, HMGB1 was idnetified to be a direct target gene of miR-381 in THP-1 cells. Conssitently, in a study about neuropathic pain, HMGB1 has been reported to be the target gene of miR-381 and invovle in its neuroprotective effect against the development of neuropathic pain [26]. Increased plasma or serum levels of HMGB1 have been found in various types of tumors, such as colon carcinoma, chronic lymphocytic leukemia and hepatocellular carcinoma, and promotes tumor progression [27]. It has been manifested that it is overexpressed in AML cell lines [28]. Furthermore, in the serum of childhood ALL patients, HMGB is reported to be highly expressed, and overexpression of HMGB stimulates leukemic cells to secrete TNF-alpha through MAPK signaling [29]. Collectively, we speculated that miR-381 might influence the proliferation of pediatric AML by targeting HMGB1 with the involvement of MAPK signaling. But its specific mechanism still needs further study. In the present study, only CCK-8 assay was performed to refeclt the cell viability, other in vitro analyses, such as cell cycle and colony-forming capacity, will improve the characterization of miR-381 on AML cell proliferation, which should be taken into account in future. Additionally, considering the dysregualtion of miR-381 in pediatric AML patients, it will be interesting to assess the expression of miR-381 in matched remission samples. But these elements were not included in the present study, which might be limitations of the study, which are worth exploring in future.

## Conclusion

In conclusion, we have confirmed for the first time the expression pattern of miR-381 in AML, and the low expression of miR-381 is associated with poor prognosis in children, and miR-381 can be used as a diagnostic biomarker for pediatric AML. In addition, low expression of miR-381 can significantly promote cell proliferation, which may be a therapeutic target for pediatric AML. The underlying mechanism of the role of miR-381 in AML awaits further elucidation. Since miRNAs acts as a network rather than individually, clinical significance of other circulating miRNAs in AML should also be evaluated in future.

## Supplementary information

**Additional file 1: Table S1**. Characteristics of the 102 patients with pediatric acute myeloid leukemia.

## Data Availability

The datasets used and/or analyzed during the current study are available from the corresponding author on reasonable request.
