# Supplementary material for: Clinical significance of dysregulation of miR-381 in pediatric acute myeloid leukemia
Source: Eur J Med Res. 2020 Sep 16;25:42. doi: 10.1186/s40001-020-00442-1 (PMC7493963; doi:10.1186/s40001-020-00442-1)
Supplement: Supplementary file 1 — Additional file 1: Table S1. Characteristics of the 102 patients with pediatric acute myeloid leukemia. [file 40001_2020_442_MOESM1_ESM.docx]

**Table S1**. Characteristics of the 102 patients with pediatric acute myeloid leukemia

| Clinical variables | No. of patients (%) |
| --- | --- |
| Age (years) |  |
| > 6 | 45 (44.12) |
| ≤ 6 | 57 (55.88) |
| Gender |  |
| Male | 52 (50.98) |
| Female | 50 (49.02) |
| WBC counts (× 10^9^/L) |  |
| >10 | 52 (50.98) |
| ≤ 10 | 50 (49.02) |
| Leukocyte (/μl) |  |
| >10,000 | 47 (46.08) |
| ≤10,000 | 55 (53.92) |
| FAB classification |  |
| M1 | 1 (0.98) |
| M2 | 38 (37.25) |
| M3 | 3 (2.94) |
| M4 | 25 (24.52) |
| M5 | 19 (18.63) |
| M6 | 4 (3.92) |
| M7 | 12 (11.76) |
| Extramedullary disease |  |
| Absent | 53 (51.96) |
| Present | 49 (48.04) |
| Cytogenetics |  |
| 11q23 | 36 (35.29) |
| Inv (16) | 13 (12.75) |
| t (8; 21) | 33 (32.35) |
| t (15;17) | 3 (2.94) |
| CN | 17 (16.67) |
| Day 7 response to treatment |  |
| Favorable | 62 (60.78) |
| Unfavorable | 40 (39.22) |

WBC, White Blood Cells; FAB, French-American-British; CN, Cytogenetically Normal.
